# A Nut-and-Bolt Microfluidic Mixing System for the Rapid Labeling of Immune Cells with Antibodies

**DOI:** 10.3390/mi11030280

**Published:** 2020-03-09

**Authors:** Jakir Hossain Imran, Jung Kyung Kim

**Affiliations:** 1 Department of Integrative Biomedical Science and Engineering, Graduate School, Kookmin University, 77 Jeongneung-ro, Seongbuk-gu, Seoul 02707, Korea; jhimran11@kookmin.ac.kr; 2 School of Mechanical Engineering and Department of Integrative Biomedical Science and Engineering, Graduate School, Kookmin University, 77 Jeongneung-ro, Seongbuk-gu, Seoul 02707, Korea

**Keywords:** nut-and-bolt microfluidics, reaction efficiency, mixing, antibody labeling, CD4+ T-cells, fluorescence imaging

## Abstract

A nut-and-bolt microfluidic system was previously developed for a point-of-care (POC) human immunodeficiency virus (HIV) test and was able to acquire images of CD4 (cluster of differentiation 4) + T-lymphocytes in a sample drop of blood followed by image analysis. However, as the system was not fully integrated with a sample reaction module, the mixing of the sample with the antibody reagent was carried out manually. To achieve a rapid reaction with a reduced amount of costly reagent in a POC diagnostic system, an efficient sample mixing function must be implemented. Here, we propose a novel method to drastically accelerate the process of sample mixing and increase the reaction rate in the nut-and-bolt microfluidic system, where the sample is mixed with the reagent in a reaction chamber formed by connecting a nut with a bolt-like sample cartridge. The mixing is facilitated by rotating the sample cartridge bidirectionally using a DC motor, which agitates the sample in a chaotic manner. A microbead complex formed by the avidin–biotin interaction was used as a model reaction system to examine the feasibility of our mixing module. We found that the reaction time for the avidin–biotin binding by mixing was 7.5 times shorter than in the incubation method, achieving a reaction efficiency of over 95%. The performance of our mixing system was further demonstrated by measuring the concentration of CD4 cells labeled with a fluorescent antibody in the blood sample. The antigen–antibody reaction mixing was faster by a factor of 20, reaching a reaction efficiency comparable to the conventional incubation method.

## 1. Introduction

The helical minichannel-based nut-and-bolt microfluidic system is one of the most effective in the field of point-of-care (POC) diagnostic systems [1,2,3]. It was developed for monitoring human immunodeficiency virus (HIV)/acquired immunodeficiency syndrome (AIDS) progression and can detect CD4 (cluster of differentiation 4) + T-lymphocytes in human blood. This system allows the scanning of a relatively large volume of a sample in a helical minichannel by rotating a cylindrical sample cartridge coupled with a single DC motor, which notably simplifies the associated electromechanical parts. 

To conform the ASSURED criteria (affordable, sensitive, specific, user-friendly, rapid and robust, equipment-free, and deliverable to end users) that were set out by the World Health Organization, microfluidic POC devices should contain all the tools for sample loading, sample mixing, and the detection of biochemical reactions. Furthermore, for microfluidic devices to be used in remote or rural regions or private clinics, they should be cheap, have low power consumption, and be easy to operate [4,5,6,7,8].

Microfluidic devices have some limitations, however. One of the challenges is mixing, which is required for labeling the samples with reagents for selective detection and further analysis. Therefore, it becomes essential in POC devices to find a way to increase the efficiency of mixing. Different types of mixing systems are available, but not all of them can create a homogeneous distribution in the fluid [9,10,11,12]. Mixing can be enhanced in centrifugal microfluidic systems using the inherent forces created by rotation [13,14,15]. Centrifugal microfluidic technologies based on unidirectional rotation create a particular flow pattern in the fluids, and particles tend to stick to the walls of the chamber as a result of the centrifugal force, which ultimately results in poor-quality mixing [16,17].

Bidirectional rotation, where the mixer alternates its rotational direction as well as oscillates in the axial direction, is an ideal solution to the above problem [18]. This motion generates sufficient turbulence for thorough mixing and results in a mixture of excellent quality. Noroozi et al. [19] proposed a micro-mixing method based on bidirectional flow. This system reduced the processing time and reagent consumption by an order of magnitude. Lin et al. [20] demonstrated a POC device in which decanting and mixing were performed by alternating the spin direction, and the process was completed within 2 s.

Although numerous studies have focused on the development of lab-on-a-chip mixing devices for improving biochemical analysis, there remains great interest in investigating novel solutions for practical applications in the field setting. In this study, we developed a nut-and-bolt microfluidic mixing module based on bidirectional motor rotation, which agitates the fluid in a chaotic manner. A feasibility study was done with an avidin-biotin binding model to confirm the enhanced reaction rate compared with the diffusion-based incubation method. We further demonstrated mixing performance by evaluating the antibody-labeled CD4 cell concentration in a blood sample in comparison with the conventional incubation method.

## 2. Materials and Methods

### 2.1. Fabrication of Sample Cartridge

**Figure 1:**
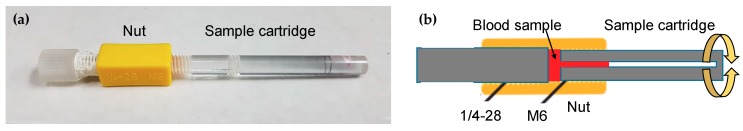
(**a**) Photograph of an assembled nut-and-bolt sample cartridge with a reaction chamber. (**b**) Cross section of a nut-and-bolt microfluidic reaction chamber formed by a nut (yellow) and a bolt-like sample cartridge (grey). The reaction chamber is filled with a blood sample (red).

As shown in Figure 1a, the mixing module comprises a nut and a bolt-like sample cartridge having an M6 screw thread at one end and a helical minichannel in the middle. We applied nut-and-bolt microfluidics for containing and mixing a sample solution in a reaction chamber, which was formed by connecting the nut and the sample cartridge, as shown in Figure 1b. The nut has an M6 thread on the inner surface, a length of 20.8 mm, and an inner diameter of 5.9 mm. The volume of the reaction chamber can be adjusted by the initial position of the sample cartridge inside the nut.

### 2.2. Sample Preparation

#### 2.2.1. Avidin–Biotin Binding Model 

In this experiment, 200 µL of microbead suspension, containing 100 µL of avidin-coated micoparticles (Straptavidin^TM^ coated microspheres, Bangs Laboratories, Fishers, IN, USA) and 2 µL of biotin-conjugated microbeads (FluoSpheres^TM^ biotin-labeled microspheres, Thermo Fisher Scientific, Waltham, MA, USA) in 98 µL of 10 × diluted lysis buffer, were mixed inside the reaction chamber. As avidin interacts with biotin through a diffusion process, it requires an incubation time of over 10 min to make them bind each other. To enhance their interaction, our newly developed automatic mixing system was applied. The microbead suspension was introduced into the reaction chamber, and the sample cartridge was rotated bidirectionally inside the nut to create chaotic advection in the sample. After completing the reaction process, 10 µL of the mixture was then taken for image analysis. All experiments including sample mixing and incubation were done at room temperature.

#### 2.2.2. Blood Sample for CD4 Count 

We collected fresh blood samples from healthy human volunteers by pricking their fingers and dispensed 100 µL of the blood sample into the reaction chamber. We then added 10 µL of CD4 fluorescent antibody (Anti-CD4 antibody [MEM-241] (Phycoerythrin), abcam, Cambridge, UK). The blood sample and the antibody were then mixed at 1000 rpm for 30 s, 1 min, and 1.5 min. Then, 10 µL of the mixed sample was taken from the reaction chamber and transferred to an imaging chamber (C-Chip, INCYTO, Cheonan-si, Chungnam-do, Korea) to obtain images that were processed and analyzed for counting the absolute number of CD4 cells.

### 2.3. Motor Control System

**Figure 2:**
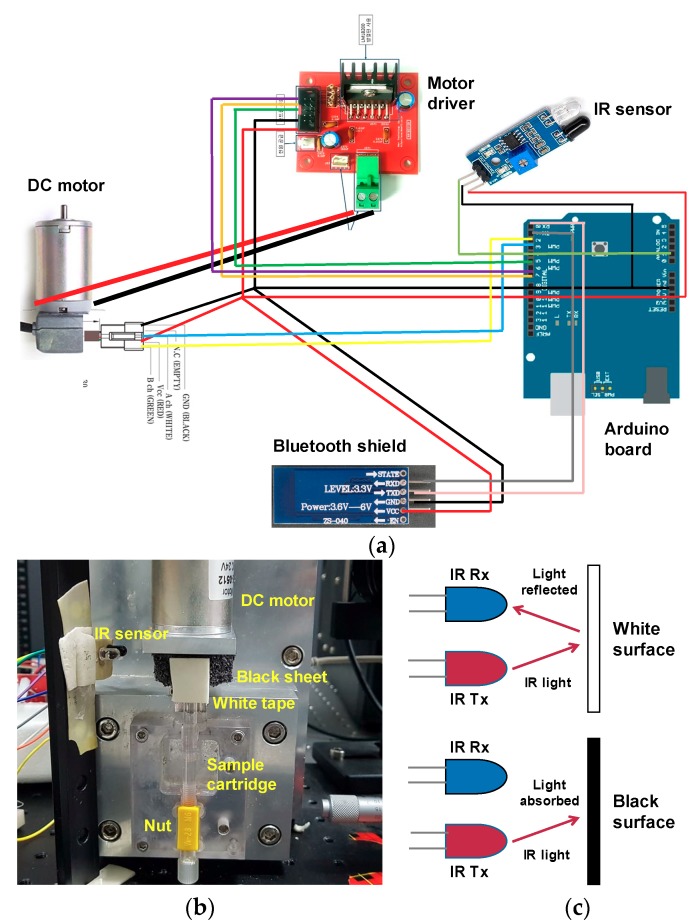
(**a**) Motor system comprising an Arduino board and a DC motor controlled wirelessly by a smartphone. (**b**) Photograph of the experimental setup for motor control system. (**c**) Schematic diagram of the working principle of the infrared (IR) sensor.

The sample cartridge was fixed to a DC motor, along with an encoder to improve the control of the rotation speed. A DC encoder motor (MB4060E-0512-24V, RobotMart, Seoul, Korea) was attached to an Arduino UNO (R3, NTREX, Incheon, Korea) board with a Bluetooth shield (HC-06, LK EMBEDDED, Seoul, Korea), so that the rotation speed and duration could be controlled using a smartphone, as shown in Figure 2a. An Arduino code was written to control the motor. We also used an infrared (IR) sensor to keep the bidirectional rotation consistent. The IR sensor has two main parts: a transmitter and a receiver. The top part of the motor was wrapped in a black sheet, and white tape was placed in a small portion of that material as shown in Figure 2b. 

As described in Figure 2c, when IR light falls onto the black surface, it is absorbed, and the receiving photodiode does not detect a signal. When IR light falls onto the white surface, it is reflected back and received by the photodiode, which generates a voltage change that is proportional to the reflectance of the surface. The receiver sends this signal to the Arduino. We tested the voltage from the receiver when IR light was reflected from the white surface and recorded this in the Arduino code. When the motor rotates, the Arduino compares the recorded voltage with the signal from the detector. When the signal matches this threshold voltage, the motor stops and changes its direction of rotation, leading to an oscillating motion. 

We controlled the motor speed by setting the pulse width modulation (PWM) speed at a 1 kHz frequency. The pulse width was calculated as (PWM/255) × 100, which varied from 0–100%. Then we converted the PWM speed into the RPM speed by multiplying the pulse width with the motor rotation speed. For 1000, 1500, and 2000 rpm, we put 35, 52, and 70 into PWM, respectively, in the motor control program. As we installed the IR sensor at one side, the motor rotated 360° in the clockwise direction and then turned counterclockwise immediately. In the motor control program, we gave the PWM speed and the number of turns in both directions as inputs. 

### 2.4. Florescence Imaging Setup

#### 2.4.1. Imaging of Microbead Complex

**Figure 3:**
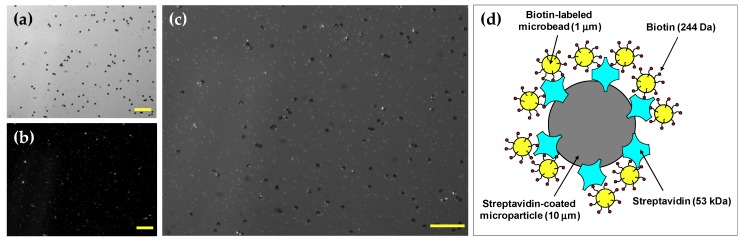
(**a**) Image of avidin-coated non-fluorescent polystyrene microparticles at the bright-field mode of the microscope. (**b**) Image of biotin-conjugated fluorescent microbeads under fluorescence illumination. (**c**) Image of avidin-biotin microbead complexes at both fluorescence and bright-field modes. (scale bar = 100 μm) (**d**) Schematic diagram of the binding of biotin-labeled microbeads to a streptavidin-coated microparticle.

After rotating the motor at 1500 rpm for a few minutes, the avidin–biotin mixture was placed onto a glass slide and images were captured using a charge-coupled device (CCD) camera (DMK21BU618, ImagingSource, Bremen, Germany) under both fluorescence and bright-field modes. The avidin molecules were conjugated with non-fluorescent polystyrene microparticles having a 10-µm diameter as shown in Figure 3a, whereas the biotin molecules were conjugated with 1-µm fluorescent microbeads as presented in Figure 3b. The fluorescent microbeads were excited at a wavelength of 505 nm and fluoresced at 515 nm. Figure 3c shows the image of microbead complexes made by the avidin–biotin interaction, which is described schematically in Figure 3d.

#### 2.4.2. Imaging of CD4 Cells

**Figure 4:**
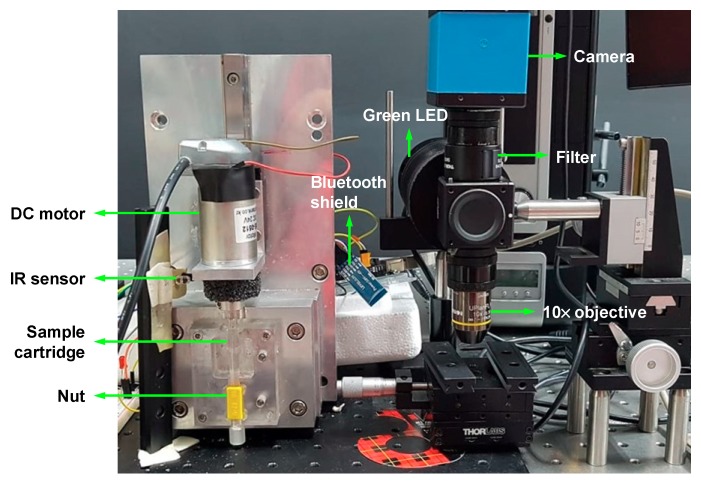
Experimental setup for nut-and-bolt microfluidic mixing and the detection system.

For the CD4 cell detection, we used a CCD camera with an excitation filter (XF108-2 Cy3, Omega Optical, Brattleboro, VT, USA) and an emission filter (595AF55 CY5M, Omega Optical, Brattleboro, VT, USA). A custom-made green LED (Boardlab, Incheon, Korea) was used as an illumination source. Our experimental setup for nut-and-bolt microfluidic mixing and the detection system is shown in Figure 4. 

The size of the image recorded was 0.6 mm in width and 0.49 mm in height, and the channel depth was 0.1 mm. Therefore, one image contained 0.0294 µL of the blood sample. The total probe volume is linearly proportional to the number of images used for analysis, which enables us to determine the cell concentration by dividing the absolute number of cells counted in the images with the total probe volume. We compared the CD4+ T-cell concentration (No. of cells per microliter) obtained by applying our mixing system with that obtained using the conventional incubation method.

## 3. Results and Discussion

### 3.1. Avidin–Biotin Reaction

A 98 μL of 10× diluted lysis buffer solution with 100-μL avidin-microparticles and 2-μL biotin-microbeads was kept in an eppendorf (EP) tube and incubated in a dark place. The sample suspensions were reacted by a diffusion process during incubation. We calculated the avidin–biotin reaction efficiency using the following formula at different incubation times:

Reaction efficiency (%) = (No. of avidin-coated microparticles bonded with biotin-labeled microbeads) × 100%/(Total No. of avidin-coated microparticles).

**Figure 5:**
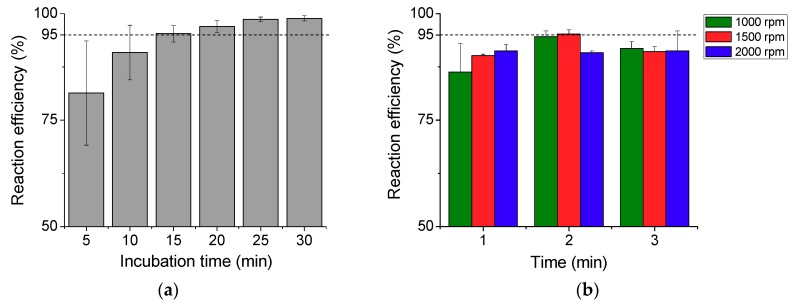
(**a**) Reaction efficiency measured at the diffusion-based reaction process for different incubation times from 5 to 30 min with an interval of 5 min. (**b**) Reaction efficiency achieved by bidirectional rotation mixing at different motor rotation speeds (1000, 1500, and 2000 rpm) and durations (1, 2, and 3 min).

The reaction efficiency measured for the diffusion-based incubation method is plotted in Figure 5a. The reaction efficiency increases with the incubation time and reaches 95% after 15 min of incubation, which is considered as a rate-limiting step in microfluidic POC devices without having an active mixing function. In an ideal POC device, a sample reaction should be made in a shorter time to ensure that the entire process from loading a sample to displaying a result can be completed within 20 min. This led us to devise a method based on the bidirectional rotation of a DC motor to enhance the reaction rate without increasing the concentration of the reagent. In this process, a fixed amount of the sample was mixed in the reaction chamber by spinning the sample cartridge connected to the DC motor, and the mixing efficiency was analyzed for different rotation speeds and durations. 

Figure 5b shows the reaction efficiency of this process. Higher efficiency was achieved for 2 min spinning time than for 1 min at both 1000 rpm and 1500 rpm, and the reaction efficiency increased with spinning time. When the motor rotated inside the mixing chamber at high speed, a small amount of sample leaked out of the chamber and this could be the reason for the lower efficiency at 3 min of spinning time. Approximately a 95% reaction efficiency was achieved within 2 min by spinning the sample cartridge bidirectionally at 1500 rpm. The bidirectional mixing method reduced the reaction time by a factor of 7.5 when compared to the incubation method for the avidin–biotin reaction. 

We compared the reaction efficiency of bidirectional rotation with unidirectional rotation. For unidirectional rotation, we rotated the motor two revolutions in one direction at a speed of 1000 rpm. For bidirectional rotation, we rotated the motor one revolution clockwise and another revolution anticlockwise, at the same rotation speed. We could not rotate the motor in one direction further as the nut-and-bolt mechanism limited the number of turns of the sample cartridge inside the nut. A slightly higher efficiency (~6%) was achieved by bidirectional rotation at this condition. Given that the duration of motor rotation was only 0.12 s, we anticipate that the difference would be more significant if we compare the reaction efficiency at longer duration up to 2 min.

### 3.2. CD4 Cell Labeling with Antibody

**Figure 6:**
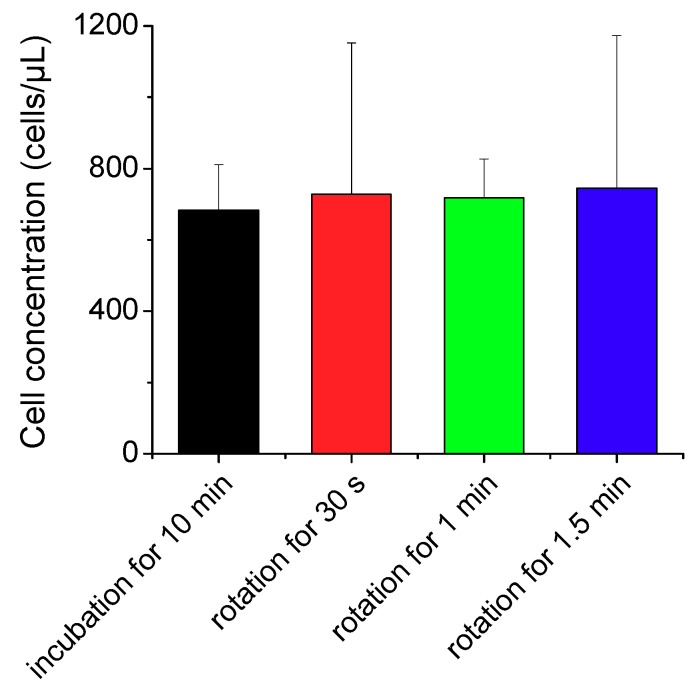
Measured concentrations of CD4 cells labeled with antibodies using the conventional incubation method and nut-and-bolt microfluidic mixing method that induces a chaotic advection within the reaction chamber by applying bidirectional rotation of the sample cartridge at 1000 rpm.

The CD4 cell images obtained were 0.6708 mm in width and 0.8867 mm in height, and the channel depth was 0.1 mm. Therefore, one image contained 0.0595 µL of the blood sample volume. We took 10 images at different parts of the imaging chamber and counted the absolute number of CD4 cells in the fixed volume to measure the CD4 cell concentration. We incubated the blood sample with CD4 antibodies for 10 min and then applied our sample mixing system at 1000 rpm for comparison of the reaction rate. Different binding characteristics between avidin-biotin and antigen-antibody reactions require different parameter sets for efficient mixing. As we obtained lower numbers of CD4 cells at 1500 rpm in our preliminary study, we performed the labeling of CD4 cell with antibody at 1000 rpm. The duration for sample mixing was set at 30–90 s to avoid the blood clotting at longer durations. As shown in Figure 6, the CD4 cell concentration was slightly higher in the mixing method than in the incubation method even with a much shorter reaction time by a factor of 20. Importantly, our results imply that the labeling of immune cells with specific antibodies can be accomplished in less than a minute using the proposed mixing module without increasing the amount of costly antibodies. 

The CD4 count is critical to measure the effectiveness of the anti-retroviral therapy (ART) in HIV infected persons. A rapid diagnostic system that enumerates CD4 cells with high accuracy and precision needs to be developed, especially for diseases prevalent in resource-constrained settings [21,22,23]. The desired technology must fulfill basic requirements including cost-effectiveness, rapidness, reliability, and accuracy [24]. Our semi-automated sample mixing and detection system has potential diagnostic applications to meet those needs, while minimizing the role of skilled technicians and reducing the chances of contamination. It can be extended to monitoring many other types of immune cells that express various CD antigens simply by labeling them with a small amount of specific antibodies.

**Figure 7:**
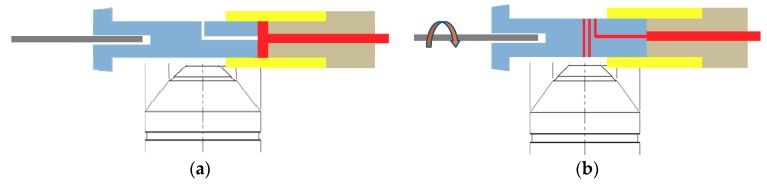
Schematic of the integrated nut-and-bolt microfluidic system for sample loading, reaction, transport, and detection. (**a**) Blood sample loaded in the mixing chamber of the sample cartridge from a microcapillary tube filled with capillary blood (blood sample is colored in red). (**b**) Sample introduced to the helical minichannel in the sample cartridge for CD4 detection by rotating the motor after the reaction process.

Although the conventional vortex can be used for sample mixing, we devised the proposed system, as the mixing module will be a part of our integrated system under development. Our ultimate goal is to develop a fully integrated microfluidic system that can perform sample loading, reaction, transport, and detection as described schematically in Figure 7. Following our previous study [1] for CD4 detection using the same sample cartridge and imaging system, we implemented a sample reaction module and enhanced reaction efficiency without additional components. After a sample is mixed inside the cartridge as demonstrated in the present study, the reacted sample can be transported from mixing chamber to a helical minichannel by rotating the motor at lower speeds and the number of CD4 cells can be detected seamlessly by acquiring and analyzing the images of fluorescently-labelled CD4 T-cells. All the procedures can be done automatically in a single platform.

With advances in microfluidic lab-on-a-chip technologies, researchers have begun to investigate mobile diagnostic platforms that can detect and analyze cells at the point of care [25,26,27,28]. Zhu et al. [28] engineered an imaging module that can be attached to a mobile phone to analyze cell images. The system comprises affordable off-the-shelf optical components that are readily obtainable. Martinez et al. [29] was the first to propose a case by using a mobile phone for telemedicine and off-site diagnosis. Since then, the number of POC test applications based on smartphones have increased because of their high visibility, data-processing capabilities, and better performance of sensors [30]. 

We ultimately aim to develop a fully integrated and automated POC system based on nut-and-bolt microfluidics, combining sample loading, transportation, mixing, and detection in a mobile platform. By employing the efficient mixing method proposed in this study, a low-cost CD4 monitoring device can be developed in the near future to replace the large and expensive analytical instruments currently being used as gold standards. The developed system would be able to provide a cost-effective and rapid solution for POC testing compared to the various existing technologies. 

## 4. Conclusions

We developed a nut-and-bolt microfluidic mixing module that facilitates the rapid labeling of immune cells in a blood sample with a specific antibody, with the aim of implementing this technique in mobile POC diagnostic platforms. Our mixing system achieved a reaction efficiency of over 95% for both avidin–biotin binding and CD4 antigen–antibody interactions with the reaction times greatly reduced by factors of 7.5 and 20, respectively, compared with the conventional incubation method. The proposed mixing module can be integrated with our cell counting system to decrease the operational time and provide rapid results, which are essential requirements for POC diagnostic devices in field settings.
